# Cytokines, Chaperones and Neuroinflammatory Responses in Heroin-Related Death: What Can We Learn from Different Patterns of Cellular Expression?

**DOI:** 10.3390/ijms141019831

**Published:** 2013-09-30

**Authors:** Margherita Neri, Laura Panata, Mauro Bacci, Carmela Fiore, Irene Riezzo, Emanuela Turillazzi, Vittorio Fineschi

**Affiliations:** 1 Department of Forensic Pathology, University of Foggia, Ospedale Colonnello D’Avanzo, Viale degli Aviatori 1, Foggia 71100, Italy; E-Mails: margheritaneri@hotmail.com (M.N.); carmelafiore@hotmail.com (C.F.); ireneriezzo@tin.it (I.R.); emanuela_turillazzi@inwind.it (E.T.); 2 Department of Forensic Pathology, University of Perugia, Via del Giochetto, Perugia 06100, Italy; E-Mails: laurapanata@gmail.com (L.P.); mauro.bacci@unipg.it (M.B.)

**Keywords:** heroin-related death, cytokines, chaperones, neuroinflammatory response, oxygen-regulated protein 150, heat shock proteins, cyclooxygenase-2

## Abstract

Heroin (3,6-diacetylmorphine) has various effects on the central nervous system with several neuropathological alterations including hypoxic-ischemic brain damage from respiratory depressing effects and neuroinflammatory response. Both of these mechanisms induce the release of cytokines, chemokines and other inflammatory mediators by the activation of many cell types such as leucocytes and endothelial and glial cells, especially microglia, the predominant immunocompetent cell type within the central nervous system. The aim of this study is to clarify the correlation between intravenous heroin administration in heroin related death and the neuroinflammatory response. We selected 45 cases among autopsies executed for heroin-related death (358 total cases); immunohistochemical studies and Western blotting analyses were used to investigate the expression of brain markers such as tumor necrosis factor-α, oxygen-regulated protein 150, (interleukins) IL-1β, IL-6, IL-8, IL-10, IL-15, cyclooxygenase-2, heat shock protein 70, and CD68 (MAC387). Findings demonstrated that morphine induces inflammatory response and cytokine release. In particular, oxygen-regulated protein 150, cyclooxygenase-2, heat shock protein 70, IL-6 and IL-15 cytokines were over-expressed with different patterns of cellular expression.

## 1. Introduction

The use of drugs for amusing intents is a remarkable issue that encompasses relevant health, judicial and forensic consequences. A broad spectrum of drugs is commonly consumed and cause severe morpho-functional impairment on all organ systems in the body. The majority of drug-related deaths are due to opiates, especially intravenously administered heroin (3,6-diacetylmorphine) that accounts for a substantial number of drug-related illnesses and injuries [1–3]. On the central nervous system, heroin has variously effects including hypoxic-ischemic brain damage from respiratory depressing effects [1,2,4,5] and neuroinflammatory response [6]. Both mechanisms induce the release of cytokines, chemokines [3,7–11] and other inflammatory mediators by the activation of many cell types such as leucocytes, endothelial and glial cells [12–14], especially microglia [7,15] which is the predominant immunocompetent cell type within the central nervous system [13–15]. A cascade of biomolecular events occurs in an intricate network after exposure to heroin [6,11–13] including the production of interleukins 1β, 6, 10 (IL-1β, IL-6, IL-10) [3,6–10,15,16], tumor necrosis factor-α (TNF-α) [3,7–10] along with the induction of cyclooxygenase-2 (COX-2) [7]. These molecules have a various set of functions [14–16]. A proinflammatory behavior is reported for TNF-α, IL-1β and COX-2, which are important mediators of acute inflammatory response [12–14,17], such as for the recruitment of neutrophil leukocytes [13]. Other molecules recognized with an immunosuppressant role [14,18] include IL-10, which inhibits cytokine production and receptor expression; additionally, it attenuates the activation of astrocytes [14,15,18]. In contrast to these findings, other researchers observed that heroin administration modulates the basic dynamics of the immune reaction, leading to an alteration in proinflammatory cytokine production with possible exposure to secondary disease [19]. Moreover molecular chaperones such as heat shock protein 70 (HSP-70) and oxygen-regulated protein 150 (ORP-150) are important for keeping the biological activity of cells, tissues, and organs from various kinds of stimulation [20]. In particular ORP-150, belonging to the heat shock protein family, is induced under hypoxic conditions and like HSP-70, its increase depends on aging [11,20].

The aim of this study is to clarify the correlation between intravenous heroin administration in heroin-related death and the neuroinflammatory response, and to investigate the expression of brain cellular markers such as TNF-α, ORP 150, IL-1β, IL-6, IL-8, IL-10, IL-15, COX-2, HSP-70, microglia marker (CD68/MAC387) and leukocyte marker (CD15), in an attempt to verify and define the role and expression of cytokines and mechanisms of cell death triggered in cases of heroin intoxication. On the basis of our experience at the Departments of Forensic Pathology of the Universities of Foggia and Perugia in the period 2006–2012, among autopsies performed for drug-related death (358 total cases), 45 cases of heroin related death were selected, and we performed immunohistochemical studies and Western blot analysis to investigate the expression of the brain markers listed above.

## 2. Results

Only some of the various antibodies tested revealed appreciable reactivity on brain tissue samples especially in brainstem, showing a significant difference in the group of heroin-related deaths and the control group as summarized in Table 1.

### 2.1. Immmunohistochemistry Results

The immunohistochemical findings and the gradation of the immunohistochemical reaction have been described with an ordinary scale and the median value has been reported.

The reactions were graded as follows: 0. (−) not expressed; 1 (+) isolated and disseminated expression; 2 (++) expression in scattered foci; 3 (+++) expression in widespread foci; 4 (++++) widespread expression.

Among the various markers tested, only a few have shown important significances in discriminating between the group of heroin-related death and the group of controls. We therefore discarded some markers, such as IL-1β, CD15, IL-8 and IL-10 because they did not provide discriminating characteristics of significance.

**Figure 1:**
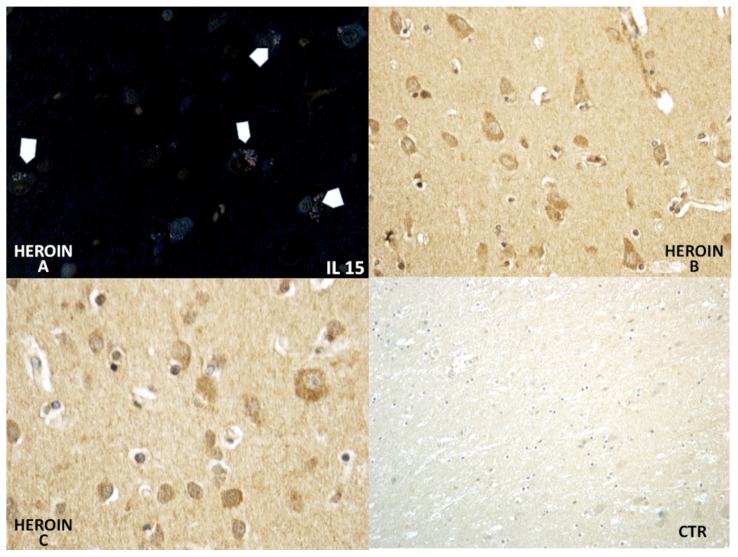
IL-15 showed a wide positive reaction in neurons, expressed by red dots in the cytoplasm [head-arrows in (**A)**]; associated to vascular (**B**); and oligodendrocyte positivity (**C**). CTR negative control case.

**Figure 2:**
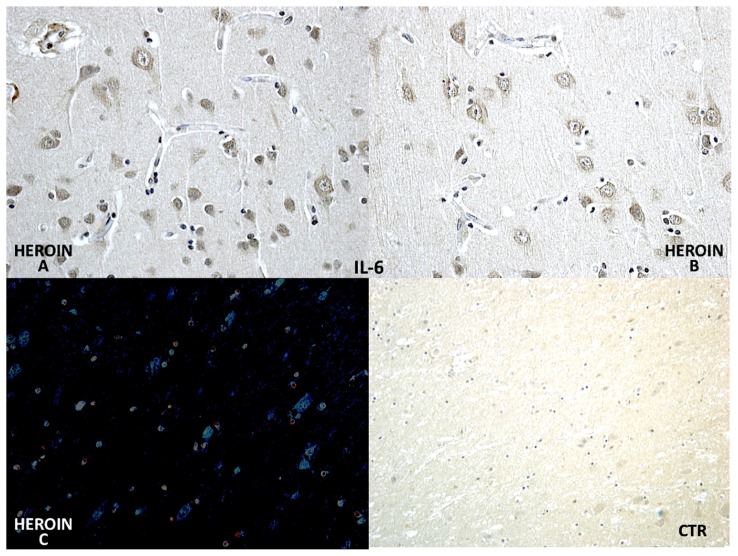
Differential immunohistochemical reaction of IL-6 in group of heroin-related death and control group showed a strong and diffusely neuronal and oligodendrocyte (**A**,**B**) positivity (in **C** confocal laser microscopy with neuronal reaction in cyan color). CTR negative control case.

Using a morphometric quantitative microscopic observation, the immunohistochemical reaction against antibodies anti-TNF-α, IL-15, MAC387 (CD68), IL-6, COX-2, HSP-70, and ORP-150 showed a statistically significant difference in the group of heroin-related death compared to the control group. Regarding cytokines IL-15 and IL-6, a wide positive reaction in neurons associated to vascular and glial positivity was observed (Figures 1 and 2).

**Figure 3:**
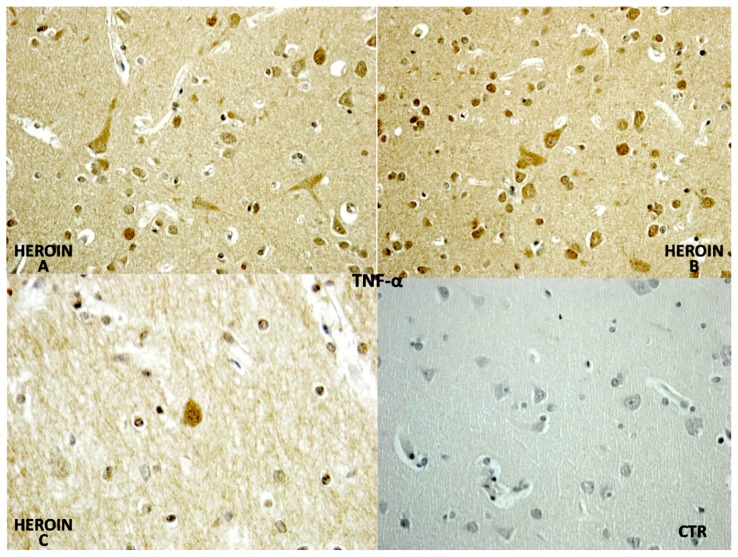
TNF-α was expressed in the heroin-related death group: note the intense neuronal and glial reactions (**A**–**C**), and the negative control group (CTR).

TNF-α reaction was intense in heroin-related death cases, with neurons and vascular localization (Figure 3).

**Figure 4:**
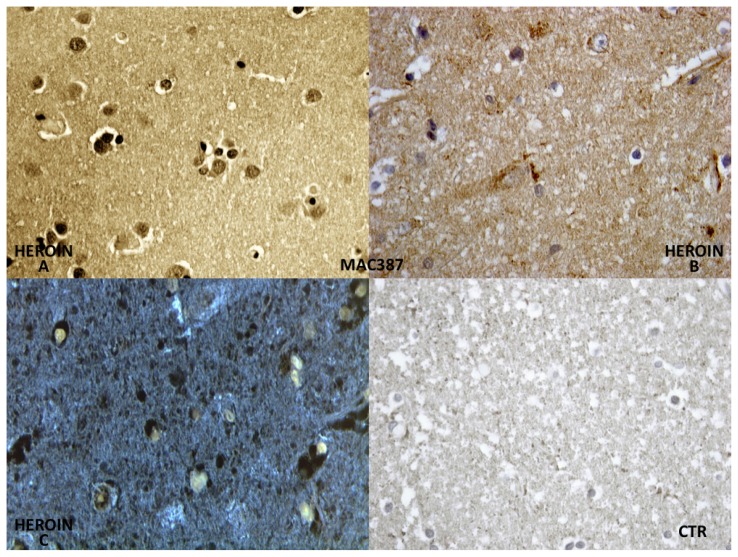
The reaction with CD68 (MAC387) showed a constant and strong positivity in brain macrophages (**A**,**B**); Evident microglia fluorescence by use of the confocal microscope (**C**); Negative control case (CTR).

Constant and strong positivity was found in brain macrophages (MAC 387) (Figure 4).

**Figure 5:**
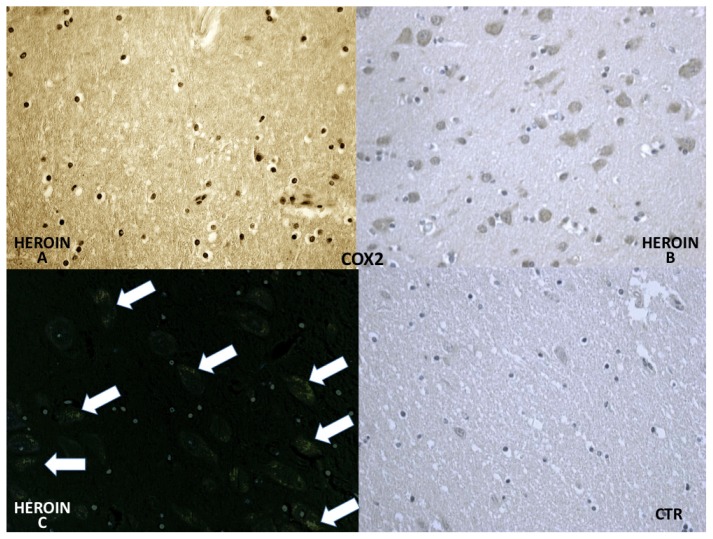
COX-2 appeared positive in glial cells (**A**) and was strongly expressed in neurons (**B**,**C**) in heroin-related death cases (confocal laser microscopy with neuronal reaction in green color). COX-2 was weakly expressed by vascular endothelium in control cases (CTR).

COX-2 reaction exhibited a strong positive reaction in the neuronal cell bodies while immunolabeling was prominent in the glial and neuronal cytoplasm (Figure 5).

**Figure 6:**
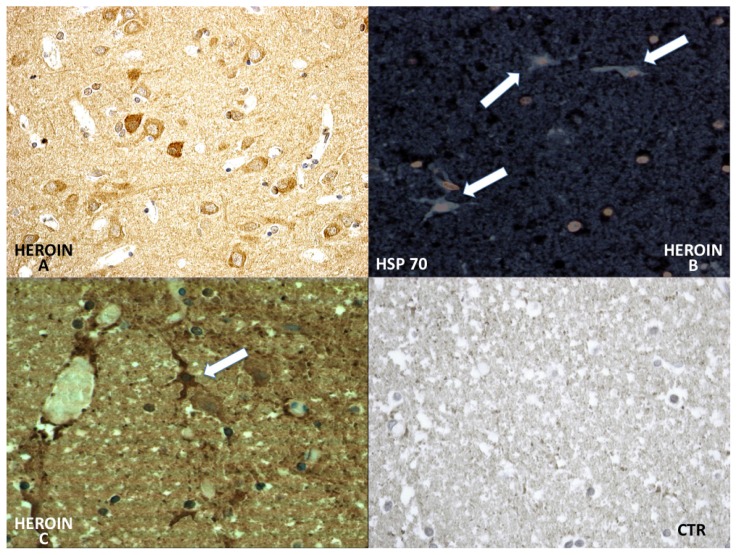
Differential immunohistochemical reaction of HSP-70: a great neuronal (**A**) involvement of HSP-70 expression was observed. The reaction was more intense in the cytoplasm of the neurons and included infrequent HSP-70 protein-positive inclusions in astrocytic fluorescence, well evidenced by use of the confocal microscope (**B**); and (**C**) The positive reaction was well appreciated also by using the bright field. Negative control case (CTR).

HSP-70 reaction was prominent in the neuronal cytoplasm of the cortical and brainstem samples (Figure 6).

**Figure 7:**
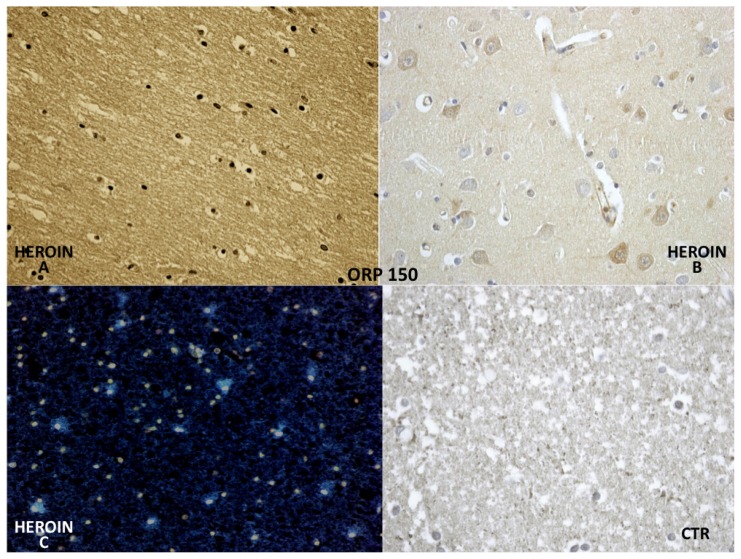
ORP-150 showing a constant microglial (**A**) and neuronal positivity (**B**); in scattered but widespread foci (cyan reaction by confocal laser microscopy in **C**); and being completely negative in the glial cells of the control group (CTR).

ORP-150 expressed an intense reaction showing a granular pattern in the cytoplasm of the neurons in the cortex and brainstem. ORP-150 was acutely expressed in neurons, strongly suggesting that expression of ORP-150 may confer neuronal resistance to early ischemic injury (Figure 7).

### 2.2. Western Blotting Results

**Figure 8:**
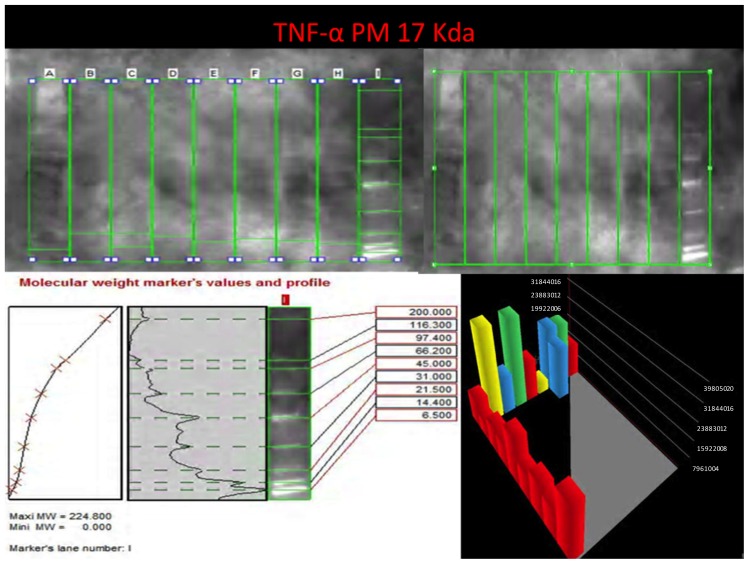
Western blot experiments with TNF-α antibodies using cytoplasmic and nuclear extracts from frozen brain of the different groups. On the left side representative blot for each group (**upper**) and TNF-α quantitative expression (**lower** and on the right). Marker’s lines on the right side of the blot lines.

**Figure 9:**
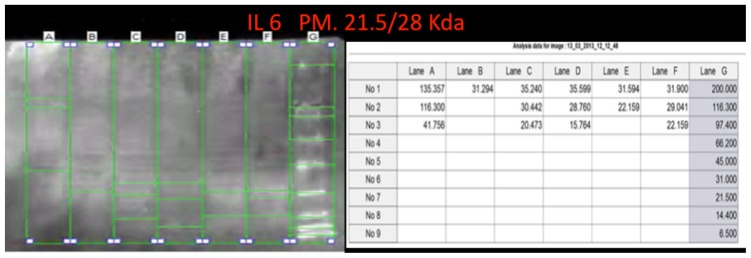

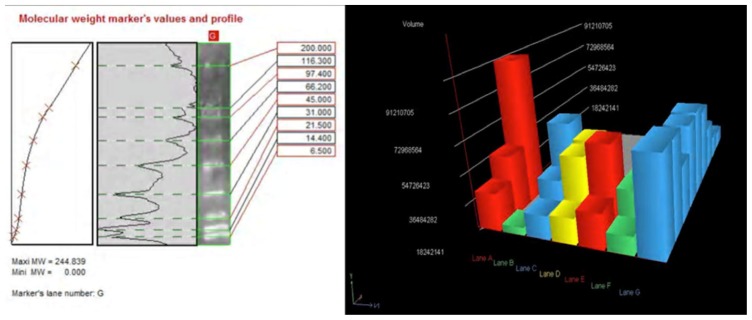
IL-6 representative blot and quantitative expression. On the right side representative blot (**upper**) and quantitative expression (**lower**). Marker’s lines on the right side of the blot lines.

**Figure 10:**
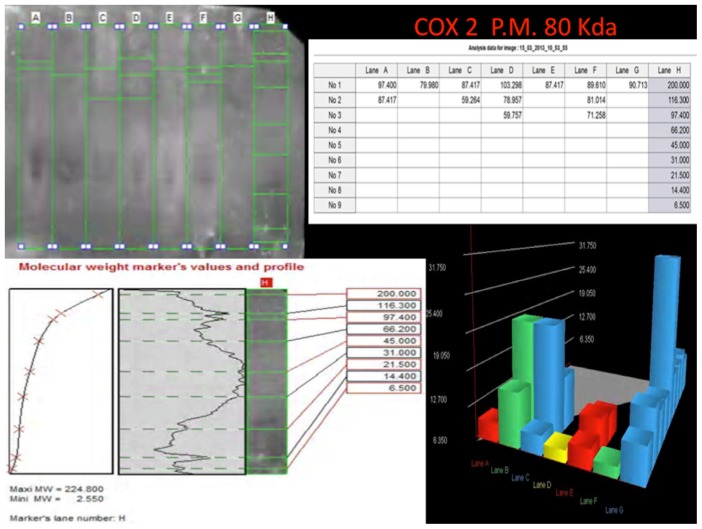
Western blotting quantitatively measured by densitometry, confirming the reactions of intense positivity for COX-2. Densitometry using cytoplasmic and nuclear extracts from frozen brain for each group, confirming the weak positivity reactions for heroin-related group. The higher result is on line (heroin group) C. Marker’s lines on the right side of the blot lines.

Western blot analysis was performed in order to confirm the results of the immunohistochemical analysis. Protein extracts from samples of frozen brain of both groups of heroin-related death and controls were used. Both cytoplasmic and nuclear extracts were prepared from the same amount of brain samples (in particular brainstem samples) and subjected to immunoblot analysis with anti-TNF-α, IL-6, COX-2. There was a perfect correspondence between immunohistochemical and blotting results, which were measured quantitatively by densitometry, confirming the strong positivity for TNF-α, IL-6, COX-2. (Figures 8–10)

## 3. Discussion

It is now well accepted that morphine induces neuroinflammatory response within the brain, but the exact etiology of the different neuropathological alterations associated with heroin abuse is still unclear and may also be related to additional substances used as adulterants [5,6].

The results obtained with immunohistochemistry and Western blotting, carried out on brain samples, in an attempt to verify and objectify the role and expression of cytokines and the mechanisms of cell death triggered in cases of heroin intoxication, raised important points and seem to provide guidelines on the interpretation of major importance questions. The immunohistochemical picture obtained using the panel of the antibodies, has shown the existence of a precise expression of the different markers in heroin-related death, which is correlated to a different stimulation of the different cellular types and also to a different response by the same cells. In our study acute cases of heroin-related death were selected, with negative toxicological analysis for other drugs, and particularly significant was the immunohistochemical study of the brain and brainstem sections. The induction of cytokines, chaperone and interleukin expression levels were confirmed by Western blot analysis on the same brain samples. This technique allowed us to quantify the expression of the inflammatory response.

In particular, chaperonin HSP-70 has provided very interesting results. It has been demonstrated that brain ischemia depletes ATP and changes intracellular homeostasis, thus disabling ATP-dependent protein quality control systems including molecular chaperones, folding enzymes, and protein degradation components during and after ischemia [11–23]. In our study HSP-70, which is not expressed in control cases, has shown a great neuronal and glial involvement and intravascular positivity of HSP-70 expression in the heroin-related death group. The reaction was more intense in the cytoplasm of the neurons and included infrequent HSP-70 protein positive inclusions in glial cells.

The reaction with ORP-150 was in agreement with those reported in the literature [24], showing a constant neuronal and glial positivity, in scattered but widespread foci, in most cases belonging to the heroin related death group and being completely negative in the glial cells of the control group. ORP-150 was acutely expressed in neurons, suggesting that strong expression of ORP-150 may confer neuronal resistance to early ischemic injury [24]. Therefore, this antigen-antibody reaction can provide useful indications about the time of onset of the hypoxic-ischemic damage.

COX-2, the protein responsible for prostaglandin synthesis, expressed by vascular endothelium in control cases, appeared more positive not only at the vascular level but also in glial cells and was strongly expressed in neurons in heroin related death cases.

In summary, our results demonstrate that molecular chaperones HSP-70 are strongly expressed in neurons and that ORP-150 expression is correlated with cell survival, indicating that induction of this stress protein may confer neuronal resistance to acute ischemic injury [24]. COX-2 is a very useful marker to detect acute neuronal damage during the early stage of focal ischemic encephalopathy [25].

The reaction with CD68 (MAC387) has shown a constant and strong positivity in microglia, so as to better identify the cells where the positive reaction is a consequence of a hypoxic-ischemic insult.

Regarding cytokines (IL-15, IL-6, TNF-α), we observed a wide positive reaction in neurons associated with vascular and glial positivity. The reaction to IL-1β, IL-8 and IL-10 was weak and without significant difference to control cases.

A wide spectrum of neuropathologic changes is present in the brain of heroin abusers. The main findings are due to infections, either due to bacterial spread from bacterial endocarditis, mycoses, or from HIV-1 infection [1–6]. Other complications include hypoxic-ischemic changes with cerebral oedema, ischemic neuronal damage and neuronal loss, which are expected to occur under conditions of prolonged heroin-induced respiratory depression, stroke caused by thrombembolism, vasculitis, septic emboli, hypotension, and positional vascular compression [24–26]. Nitric oxide (NO) and its metabolites are described to be involved in cell physiological and pathological events related to heroin intoxication, mainly resulting from increased activity of inducible nitric oxide synthase (iNOS) in polydrugs abusers [27]. Recently, findings from the literature showed that neuronal nitric oxide synthase (nNOS) activity might also be involved, resulting in high brain NO levels [28].

One of the causes of death in heroin addiction is respiratory failure, often accompanied by pulmonary complications, especially oedema. The death is generally due to severe acute poisoning, with regard to the degree of opioid tolerance possessed by the subjects at the time of the lethal dose.

As Büttner stated [5], at autopsy, up to 90% of all cases of heroin-related death show brain oedema with prominent tonsillar herniation and uncal grooving. However, rapid death after heroin intake has no morphological evidence of cell injury. A survival period of more than 5 h could lead to hypoxic-ischaemic encephalopathy with loss of neurons in the hippocampal formation, the Purkinje cell layer and/or the olivary nucleus, as well as vascular congestion. It is extremely difficult to distinguish whether the neuronal alterations may be related to hypoxic episodes during a state of intoxication induced by heroin or if they are caused by the direct toxic effects of the toxic substance [2,21,22].

## 4. Experimental Section

The toxicological data and the autopsy records of the 358 autopsies of drug related-death performed at the Departments of Forensic Pathology of the University of Foggia and of the University of Perugia (Perugia, Italy) over the period 2006–2012 were evaluated, and 45 cases of heroin-related death were selected (42 men, 3 women). Post-mortem delay interval 20.5 ± 13 h. We selected only the cases with toxicological data positive only for heroin and negative for other drugs (ethanol included), and postmortem examination confirmed diagnosis of heroin-related death. The median blood morphine concentration of cases [as determined by gas chromatography-mass spectrometry (5977A Series Agilent, Palo Alto, CA, USA)] was 5.8 ± 0.72 mg/L (range 0.30–10.45 mg/L). All cases were HIV-1 negative. The control group was composed of 45 cases selected among traumatic deaths, without brain lesions and with negative toxicological analysis for drugs. Standard sample blocks were taken from the cerebral cortex, white and grey matters, basal ganglia, thalami and the brainstem. In detail, all lobes of the brain, central nuclei (caudate, putamen, pallidum and thalamus), cerebellum, pons and medulla oblongata were included. In each case, the tissue samples were fixed in 10% formalin for 48 h and then processed and embedded in paraffin. For each case, total sections of about 4 μm thickness were cut and stained with haematoxylin and eosin (H&E). In addition, immunohistochemical investigation of all samples was performed utilizing a panel of antibodies IL-6 (Santa Cruz, CA, USA), IL-8, (Abcam, Cambridge, UK), IL-10 (Peprotech, London, UK), TNF-α (Santa Cruz, CA, USA), CD15 (DAKO, Copenhagen, Denmark), IL-1β (Santa Cruz, CA, USA), IL-15 (R & D Systems, Minneapolis, MN, USA), HSP-70 (NovaCastra, Milton Keynes, UK), ORP-150 (IBL, Fujioka Goiania, Japan), COX-2 (Santa Cruz, CA, USA), CD68/MAC387 (Serotec, Oxford, UK). We used 4 μm thick paraffin sections mounted on slides covered with 3, amminopropyl-triethoxysilane (Fluka, Buchs, Switzerland). Pre-treatment was necessary to facilitate antigen retrieval and to increase membrane permeability to antibodies anti-CD 15, IL-1β, L-15, HSP 70, IL-8 boiling in 0.25 M EDTA buffer, to antibodies anti-TNF-α and ORP-150 boiling in 0.1 M citric acid buffer, to antibody anti-IL-6, IL-10, COX-2, CD68/MAC387 for 15 min in Proteolytic Enzyme (Dako, Copenhagen, Denmark), at 20 °C.

The primary antibody was applied in a 1:50 ratio CD-15, in a 1:4000 ratio IL-1β and IL-10, in a 1:2000 ratio IL-6, in a 1:600 ratio TNF-α, in a 1:100 ratio IL-15, COX-2 and HSP-70, in a 1:500 ratio IL-8, in a 1:200 ratio ORP-150 and CD68/MAC387 and incubated for 120 min at 20 °C. The detection system utilized was the LSAB + Kit (Dako, Copenhagen, Denmark), a refined avidin–biotin technique in which a biotinylated secondary antibody reacts with several peroxidase conjugated streptavidin molecules. The positive reaction was visualized by 3,3-diaminobenzidine (DAB) peroxidation, according to standard methods. The sections were counterstained with Mayer’s haematoxylin, dehydrated, coverslipped and observed in a Leica DM4000B optical microscope (Leica, Cambridge, UK). The samples were also examined under a confocal microscope, and a three-dimensional reconstruction was performed (True Confocal Scanner; Leica TCS SPE, Milan, Italy,). A preliminary semi-quantitative evaluation of the immunohistochemical findings was made by two different investigators without prior knowledge. The reactions were graded as follows: 0 (−) not expressed; 1 (+) isolated and disseminated expression; 2 (++) expression in scattered foci; 3 (+++) expression in widespread foci; 4 (++++) widespread expression. All measurements were done using the same magnification of image (10×) and by the same two examiners. A third blind microscopic evaluator was involved to weigh the histological evidences.

### 4.1. Western Blot Analysis

Western blot analysis was performed. Approximately 100 mg of brainstem frozen tissue was dissected and immediately transferred to RIPA buffer with protease inhibitor cocktail and homogenized on ice utilizing homogenizer Silent Crusher. The homogenate was centrifuged (12000 rpm for 10 min at 4 °C). The supernatant was collected, estimated by Qubit Fluorometer (Invitrogen, Carlsbad, CA, USA), and boiled for 5 min, at 95 °C. Brain total protein extracts (approximately 40 μg/lane) were run on 4%–15% SDS PAGE at 80 V for about 2.5 h. For Western blot, proteins from SDS gels were electrophoretically transferred to nitrocellulose membranes in mini trans blot apparatus (1 h at 250 mA). Non-specific binding was blocked by incubating membranes in Western blocker solution for 1 h at room temperature. The membranes were incubated with primary antibodies, selected among the antibody with a good statistical difference between heroin related death and controls: anti-TNF-α, IL-6, COX-2 diluted in Western blocker solution, in 1:400 ratio overnight at 4 °C. Blots were washed with (phosphate buffered saline) PBS/Tween-20 and then incubated for 1 h at room temperature with (horseradish peroxidase) HRP-conjugated secondary antibodies diluted in Western blocker solution, in 1:2000 ratio. Membranes were washed with PBS/Tween-20, and the immune reaction was developed in IMMUNOSTAR Kit Western C (Bio-Rad laboratories, Hercules, CA, USA) and then visualized by Chemiluminescent detection methods. The light was then detected by photographic film. The image was analyzed by Uvitec (Cambridge, UK), which detects the chemiluminescent blots of proteins staining.

### 4.2. Statistical Analysis

Values are presented as means SD (standard deviation). The unpaired two-way Student’s *t*-test was used to compare the results obtained for heroin-related death group with the control group. *p* < 0.05 was accepted as indicative of significant difference among groups.

## 5. Conclusions

In conclusion, our findings demonstrated that morphine induces the inflammatory response and cytokine release. Deficient or excess expression of these key mediators may predispose the abusers to aberrant defence mechanisms [3]. The international scientific panorama is actually rich with studies aimed to identify markers which can provide more and more accurate information about the time of onset of hypoxic-ischemic brain damage and inflammatory response in heroin-related death. However, data reported by the literature appear fragmentary and contradictory, often concerning experimental studies performed only on animals [29,30]. The histological study of the brain with traditional histochemical techniques can provide relevant data since it is well known that depending on mechanism, severity, and timing of the insult, the distribution and the histological pattern of lesions in the brain changes dramatically. By means of immunohistochemical techniques applied in studies both on animals and humans, it has been possible to identify in the brain tissue some markers of inflammatory response with reliable and reproducible results. Furthermore, Western blotting matched perfectly with the results of immunohistochemistry.

The complex interaction mechanisms between hypoxia and/or inherent toxicity in heroin-related cases, may thus be better clarified on the basis of the reaction of the inflammatory response and cytokine cross-talk. Some immunohistochemical markers have been shown to be more reliable than others in the evaluation of inflammatory brain pattern. In particular, HSP-70, ORP-150, COX-2 reaction, and TNF-α, IL-15 and IL-6 cytokines, have provided very interesting results. Future studies should be conducted to investigate the mechanisms of action by which cytokines, chaperones and inflammatory responses are activated by heroin abuse.

## Figures and Tables

**Table 1:** Semi-quantitative evaluation and statistical analysis of the immunohistochemical findings and gradation of the immunohistochemical reaction in the brain samples.

Antibody	Group 1 heroin related-death	Group 2 controls	Statistical value group 1 *vs.* group 2
IL-1β	−	−	NS
IL-15	++++	−	***
CD15	−	−	NS
MAC387 (CD68)	+++	−	**
IL-8	−	−	NS
IL-10	+	−	NS
TNF-α	++++	−	***
IL-6	++++	−	***
COX-2	++++	−	***
HSP-70	+++	−	**
ORP-150	+++	−	**

NS: *p* > 0.05;

** : *p* < 0.01;

*** : *p* < 0.001.

Intensity of immunopositivity was assessed semiquantitatively in the scale 0–4 as follows: −: no immunoreactivity (0%); +: mild immunopositivity in scattered cells (10%); ++: immunopositivity in up to one third of cells (33%); +++: immunopositivity in up to one half of cells (50%) and ++++: strong immunopositivity in the majority or all cells (100%).
